# Lichen planus pigmentosus associated with tamoxifen: clinical presentation and pathophysiological insights^؆^

**DOI:** 10.1016/j.abd.2026.501432

**Published:** 2026-08-07

**Authors:** Luis Daniel Pérez Cáceres, María Paula Hurtado, Sergio Andrés Amaya Peña, Juan Felipe Ochoa Bermúdez

**Affiliations:** a Fundación Universitaria Sanitas, Bogotá, Colombia; b Department of Dermatology, Centro Dermatológico Federico Lleras Acosta, Bogotá, Colombia

Dear Editor,

Lichen Planus Pigmentosus (LPP) is a variant of Lichen Planus (LP), characterized by hyperpigmented macules, chronic progression, and a predilection for photoexposed areas.^1^ Its etiology is multifactorial and, in some cases, it has been associated with medication use.^2^, ^3^ Although tamoxifen is widely used in oncology and frequently linked to melasma, its association with LPP has been described only in exceptional cases.^4^ While the exact pathogenesis of tamoxifen-induced LPP remains uncertain, it is hypothesized to involve cell-mediated cytotoxic immune response, leading to interface dermatitis and subsequent pigmentary incontinence.^5^ This report describes a case of LPP associated with tamoxifen use, with an emphasis on the underlying pathophysiological mechanisms and relevant therapeutic considerations.

We report the case of a 60-year-old woman with a history of ductal breast carcinoma treated with quadrantectomy followed by adjuvant therapy with tamoxifen, who developed hyperpigmented macules on the malar region approximately one month after initiation of treatment. The lesions were initially accompanied by pruritus and progressively became more pronounced, disseminating in a photoexposed distribution. Clinically, the macules exhibited a characteristic grayish-blue coloration, suggesting dermal melanin deposition, which contributed to distinguishing this condition from melasma, the main differential diagnosis. Pigmentation of the eyelids was also observed, further supporting this distinction. The pruritus gradually subsided but recurred intermittently during treatment. Dermoscopic examination revealed thick grayish-blue perifollicular globules without obliteration of follicular openings by pigment; these findings, in contrast to those observed in melasma and exogenous ochronosis, supported our suspicion of a lichenoid drug reaction. After discontinuation of the suspected causal medication, the lesions became asymptomatic; however, persistent hyperpigmentation persisted (Fig. 1). The patient denied the use of high-concentration hydroquinone or other depigmenting agents.

**Figure 1:**
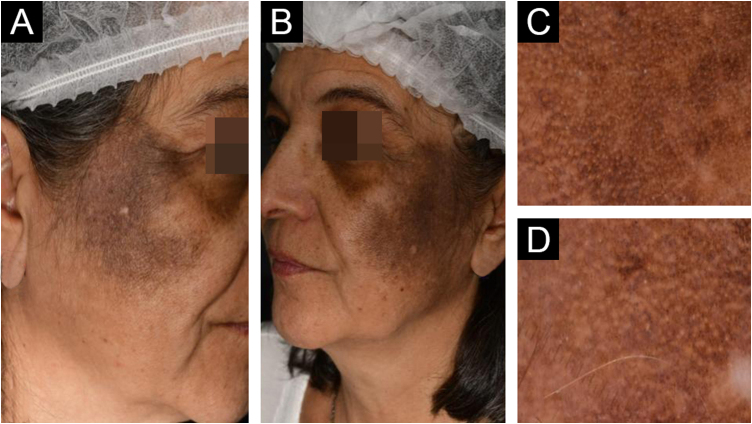
Extensive, confluent dark brown hyperpigmented macules arranged in a photo-distributed pattern. (A and B) Bilateral involvement of the malar region, including the zygomatic and periocular areas, with extension toward the cheeks. (C and D) Dermoscopic image: Presence of perifollicular coarse gray-blue globules, without obliteration of follicular openings by pigment.

Given the chronic progression of the lesions, two skin biopsies were performed. The first revealed vacuolar degeneration of the basal layer, apoptotic keratinocytes, pigmentary incontinence, and a periannexial and perivascular lymphocytic infiltrate. A second biopsy, corresponding to a later stage of the disease, demonstrated no significant inflammatory changes but revealed residual pigmentary alteration. These findings were interpreted as post-inflammatory hyperpigmentation secondary to prior interface dermatitis (Fig. 2). The clinical chronology, histopathologic evolution, and temporal association with tamoxifen supported the diagnosis of drug-induced LPP.

**Figure 2:**
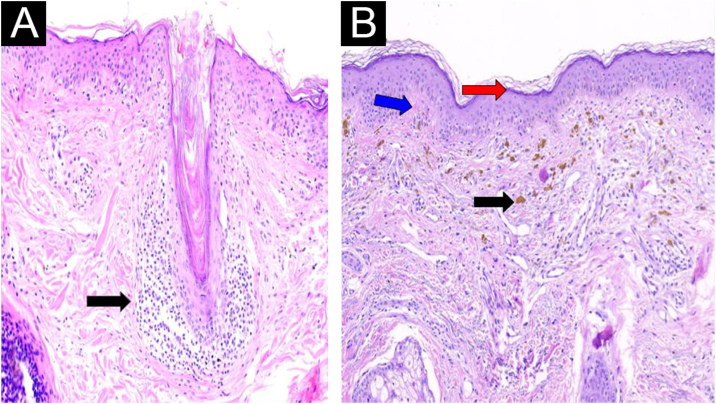
Skin biopsy stained with hematoxylin and eosin. (A) 40× magnification, corresponds to an earlier stage of the process. A mononuclear lymphohistiocytic infiltrate is observed following the follicular epithelium (black arrow). No interface damage, mucin deposition, or significant epidermal alterations are identified. These findings are suggestive of follicular LP. (B) 10× magnification, represents a later stage, characterized by postinflammatory pigmentary changes. Epidermal atrophy (red arrow), interface alteration (blue arrow), pigment incontinence (black arrow), and prominent melanophages (black arrow) are evident. Clinicopathological correlation supported the diagnosis of LPP.

LPP is an inflammatory dermatosis mediated by CD8+ T-lymphocytes targeting basal keratinocytes.^5^ The cytotoxic activity results in apoptosis of basal keratinocytes, basement membrane damage, and subsequent pigmentary incontinence.^5^ Clinically, this presents as gray-brown macules, a consequence of the Tyndall effect produced by melanin located in the upper dermis.^6^ A variety of triggering factors have been identified in the literature, as outlined in Table 1.^1^, ^2^ In the present case of tamoxifen-induced LPP, the drug may have functioned as an immunogenic stimulus, modifying antigen processing and presentation, thereby triggering a T-cell-mediated inflammatory reaction against epithelial cells.^7^

**Table 1:** Reported triggers and associated factors in the pathogenesis of LPP.^2^, ^3^

General Characteristic	Detailed Criteria
Oil-Based Compounds	Almond oil
	Mustard oil
Cosmetics	Hair dyes
	Fragrance-containing products
	Deodorants
	Cosmetic creams
	Aftershave lotions
Medications	Antimicrobials: ketoconazole, tetracyclines, griseofulvin
	Antihypertensives: captopril, labetalol, nifedipine
	Antimalarials: chloroquine, hydroxychloroquine, quinacrine
	Diuretics: furosemide, spironolactone, hydrochlorothiazide
	Non-Steroidal Anti-Inflammatory Drugs (NSAIDs): ibuprofen, naproxen, acetylsalicylic acid
	Tumor Necrosis Factor-Alpha (TNF-α) inhibitors: infliximab, adalimumab, etanercept

While tamoxifen exerts anti-estrogenic effects on breast tissue, it can display partial estrogenic activity in other tissues such as the skin.^8^ Such estrogenic effects have been linked to melasma-like hyperpigmentation, a pattern also reflected in this patient’s histopathology. Nonetheless, lichenoid tissue reactions are not typically included among the recognized adverse effects of tamoxifen.^4^

In addition to the distinctive pigmentation pattern, the photoexposed distribution observed clinically implies that ultraviolet radiation may potentiate the underlying inflammatory process. The early pruritic phase is consistent with active immune-mediated inflammation, while the subsequent symptom resolution marks a transition to a chronic stage dominated by pigmentary incontinence.^1^, ^2^, ^5^ These findings were consistent with the patient’s histopathological results.

In a patient presenting with recent-onset blue-gray macules, the clinician is faced with a broad differential diagnosis that includes melasma and the spectrum of acquired dermal macular hyperpigmentation, encompassing LPP, ashy dermatosis/erythema dyschromicum perstans, and Riehl’s melanosis with pigmented contact dermatitis. These conditions show substantial clinical, histopathological, and dermoscopic overlap, and current literature indicates that no single feature reliably distinguishes them, with many traditionally described differences being largely historical or distribution-based. Within this spectrum, Riehl’s melanosis and pigmented contact dermatitis are more often associated with cosmetic, fragrance, and hair dye exposure, typically involving the face, whereas ashy dermatosis/erythema dyschromicum perstans more commonly shows truncal involvement, occasionally with a peripheral erythematous border. LPP is more frequently associated with lichenoid inflammatory changes and prominent pigmentary incontinence, although these findings are not specific. Taken together, these clinical patterns may assist in diagnostic reasoning across this spectrum of disorders.^9^

Therefore, recognition of the biphasic pathogenesis of LPP ‒ characterized by an early immune-mediated inflammatory stage and a subsequent pigmentary phase ‒ provides insight into its immunopathology and supports the development of targeted, phase-specific treatment strategies. Management of LPP should be phase-specific: the initial inflammatory stage warrants topical or systemic anti-inflammatory therapies, while the chronic pigmentary stage necessitates interventions targeting hyperpigmentation.^5^

Although LPP typically follows a slow and chronic progression, its prognosis is cautious, given the depth of melanin deposition and permanent alteration of the dermoepidermal junction, which rarely allows for complete clinical resolution.^10^

This report highlights the need to consider drug-induced LPP as a differential diagnosis in patients with facial hyperpigmentation and a history of exposure to immunomodulatory agents like tamoxifen. Recognition of these immunopathological mechanisms is essential for diagnostic precision and the implementation of targeted therapeutic strategies.

## ORCID ID

Luis Daniel Pérez Cáceres: 0000-0003-0851-8894

María Paula Hurtado: 0009-0009-9715-6178

Sergio Andrés Amaya Peña: 0000-0002-1310-9836

## Research data availability

Does not apply.

## Financial support

The authors declare that no financial support or funding was received for this study.

## Authors’ contributions

Luis Daniel Pérez Cáceres: Approval of the final version of the manuscript; critical literature review; data collection, analysis and interpretation; effective participation in research orientation; intellectual participation in propaedeutic and/or therapeutic management of studied cases; manuscript critical review; preparation and writing of the manuscript.

Juan Felipe Ochoa Bermúdez: Approval of the final version of the manuscript; critical literature review; data collection, analysis and interpretation; effective participation in research orientation; intellectual participation in propaedeutic and/or therapeutic management of studied cases; manuscript critical review; preparation and writing of the manuscript.

María Paula Hurtado González: Approval of the final version of the manuscript; critical literature review; data collection, analysis and interpretation; effective participation in research orientation; intellectual participation in propaedeutic and/or therapeutic management of studied cases; manuscript critical review; preparation and writing of the manuscript.

Sergio Andrés Amaya Peña: Approval of the final version of the manuscript; critical literature review; data collection, analysis and interpretation; effective participation in research orientation; intellectual participation in propaedeutic and/or therapeutic management of studied cases; manuscript critical review.

## Conflicts of interest

None declared.
